# Highly selective electrochemical fluorination of dithioacetal derivatives bearing electron-withdrawing substituents at the position α to the sulfur atom using poly(HF) salts

**DOI:** 10.3762/bjoc.11.12

**Published:** 2015-01-19

**Authors:** Bin Yin, Shinsuke Inagi, Toshio Fuchigami

**Affiliations:** 1 Department of Electronic Chemistry, Tokyo Institute of Technology, Nagatsuta, Midori-ku, Yokohama 226-8502, Japan

**Keywords:** anodic fluorination, anodic fluorodesulfurization, electrosynthesis, fluorination product selectivity, poly(HF) salt

## Abstract

Anodic fluorination of dithioacetals bearing electron-withdrawing ester, acetyl, amide, and nitrile groups at their α-positions was comparatively studied using various supporting poly(HF) salts like Et_3_N·*n*HF (*n* = 3–5) and Et_4_NF·*n*HF (*n* = 3–5). In the former two cases, the corresponding α-fluorination products or fluorodesulfurization products were obtained selectively depending on supporting poly(HF) salts used. In sharp contrast, in the latter two cases, fluorination product selectivity was strongly affected by the electron-withdrawing ability of α-substituents: A dithioacetal bearing a relatively weak electron-withdrawing amide group provided a fluorodesulfurization product selectively while a dithioacetal having a strongly electron-withdrawing nitrile group gave the α-fluorination product predominantly regardless of the poly(HF) salts used.

## Introduction

The introduction of fluorine atom(s) into organic molecules very often improves or enhances their desired characteristic physical and biological properties hence organofluorine compounds are highly useful for medicinal, agrochemical, and materials science [1–6]. In order to prepare new fluorine compounds, selective fluorination of organic compounds is becoming significantly important. Although the selective fluorination has been extensively studied, highly efficient and safe fluorination methods are still demanded [7–8]. With these facts in mind, we have developed a selective electrochemical fluorination using ionic liquid poly(HF) salts such as Et_3_N·*n*HF and Et_4_NF·*n*HF (*n* = 3–5) as supporting electrolyte and fluorine source [9–11], and we have systematically studied the anodic fluorination of various heteroatom-containing compounds including heterocycles and macromolecules so far [12–20].

**Scheme 1:**
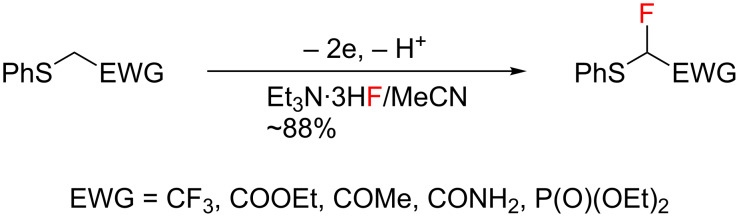
Anodic fluorination of sulfides having an electron-withdrawing group.

**Scheme 2:**
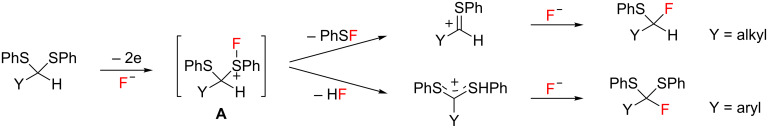
Anodic fluorination of dithioacetals.

More than 20 years ago, we reported the first successful example of the electrochemical selective fluorination of heteroatom-containing compounds such as α-(phenylthio)ester and its analogues as shown in Scheme 1 [21–22]. Furthermore, anodic fluorodesulfurization of dithioacetals was achieved by direct and indirect anodic oxidation in the presence of the poly(HF) salts [12–16,23–25] or alkali-metal fluorides like KF and CsF with PEG 200 [17]. The anodic fluorination of a dithioacetal derived from an aliphatic aldehyde provided the fluorodesulfurization product while a dithioacetal derived from an aromatic aldehyde provided the α-fluorination product (Scheme 2) [25]. These results suggest that the fluorinated product selectivity seems to be controlled by the easiness of the deprotonation of the cationic intermediate **A**. Namely, since the α-proton of the aromatic dithioacetal is more acidic compared to that of an aliphatic dithioacetal, the deprotonation of the former is faster than it is for the latter. Therefore, it can be stated that the product selectivity is controlled by the kinetic acidity of the cationic intermediate **A** [26–27].

With these facts in mind, we studied comparatively the anodic fluorination of dithioacetal derivatives having various electron-withdrawing groups at their α-positions using various poly(HF) salts [28].

## Results and Discussion

Various α-substituted methyl phenyl sulfides, **1a**, **1c**, **1e**, and **1g**, and their α-phenylthio derivatives (dithioacetals) were prepared, and their oxidation potentials (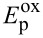) were measured by cyclic voltammetry in an anhydrous acetonitrile (MeCN) solution containing *n*-Bu_4_NBF_4_ (0.1 M) using a platinum disk working electrode and a saturated calomel electrode (SCE) as the reference electrode. All compounds exhibited irreversible multiple oxidation peaks and the first oxidation peak potentials are summarized in Table 1. It was expected that the introduction of an additional phenylthio group to the α-position of the sulfides would decrease their oxidation potentials. However, unexpectedly they are higher than those of the corresponding sulfides having a single phenylthio group. Although a detailed reason is not clear at present, the additional phenylthio group does not act as an electroauxiliary, but acts as an electron-withdrawing group. As shown in Table 1, the oxidation potentials of sulfide **1g** and dithioacetal **1h** having a stronger electron-withdrawing cyano group (Taft σ* = +1.30) [29] are much higher compared to those of **1a**, **1b** with an ester group (Taft σ* = +0.69) [29] and **1c**, **1d** with an acetyl group (Taft σ* = +0.60) [29], respectively. This indicates that the polar effect, namely electron-withdrawing effect of a substituent greatly affects the electron-transfer step from the substrate to the anode.

**Table 1:** First oxidation potentials, 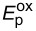 of compounds **1**.

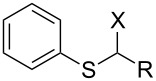			

Substrate	X	R (σ* value)^a^	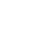 (V vs SCE)^b^

**1a**	H	COOEt (+ 0.69)	1.60
**1b**	SPh	COOEt (+ 0.69)	1.73
**1c**	H	COMe (+ 0.60)	1.59
**1d**	SPh	COMe (+ 0.60)	1.63
**1e**	H	CONEt_2_	1.60
**1f**	SPh	CONEt_2_	1.64
**1g**	H	CN (+ 1.30)	1.85
**1h**	SPh	CN (+ 1.30)	2.04

^a^From [29]. ^b^Substrate concentration: 5 mM; sweep rate: 100 mV/s; working electrode: Pt disk (Ø = 1 mm).

At first, anodic fluorination of ethyl α,α-bis(phenylthio)acetate (**1b**) [30–31] was carried out at platinum plate electrodes in an undivided cell using various solvents in the presence of Et_3_N·3HF as supporting salt and fluorine source. A constant current was passed until the starting material **1b** was completely consumed (monitored by TLC). As shown in Table 2, the anodic fluorination of **1b** proceeded to give the corresponding α-fluoro product **2b** in a good yield regardless of the solvent used. Thus, it was found that the solvents did not affect the yield of **2b** in contrast to the current efficiency. When the reaction was performed in DME as the solvent, anodic decomposition of DME took place simultaneously with the anodic fluorination of **1b**, which resulted in low current efficiency. In all cases, fluorodesulfurization product **3b** [22] was detected in considerable yield. When CH_2_Cl_2_ and MeNO_2_ were used, a small amount of ethyl α,α-difluoro-α-(phenylthio)acetate (**4b**) [22] was also detected (Table 2, entries 3 and 4). As a blank test, the electrolytic solution of **1b** was mechanically stirred without electrolysis overnight and **1b** was mostly recovered (Table 2, entry 5). Therefor electrolysis is necessary for the fluorination to take place.

**Table 2:** Anodic fluorination of **1b** in various solvents containing Et_3_N·3HF^a^.

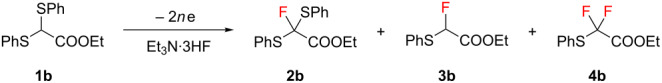						

Entry	Solvent	Charge passed (F/mol)	Yield (%)^b,c^			Total yield (%)

			**2b**	**3b**	**4b**	

1	MeCN	3.0	74 (70)	9	0	83
2	DME	5.0	74	9	0	83
3	CH_2_Cl_2_	2.5	73	5	4	82
4	MeNO_2_	2.2	73	7	1	81
5^d^	MeCN	–	–	–	–	–

^a^Constant current (8 mA/cm^2^) electrolysis was carried out in 0.3 M Et_3_N·3HF/solvent. ^b^Determined by ^19^F NMR. ^c^Isolated yield given in parentheses. ^d^Mechanical stirring was performed overnight at ambient temperature without electrolysis.

Among the solvents tested for electrolysis, we decided to use MeCN for further studies on the anodic fluorination based on a good current efficiency and the formation of only one byproduct.

**Figure 1:**
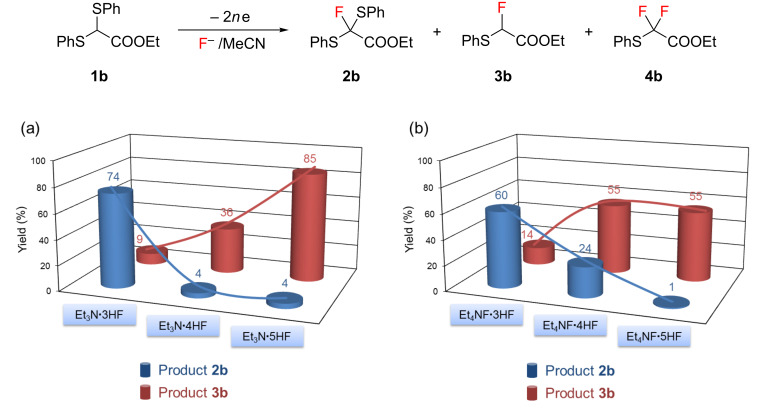
Dependency of fluorinated product selectivity on a series of fluoride salts (a) Et_3_N·*n*HF (*n* = 3–5) and (b) Et_4_NF·*n*HF (*n* = 3–5).

Next, anodic fluorination of **1b** was carried out in MeCN using various poly(HF) salts until the substrate was completely consumed and the results are shown in Figure 1. As mentioned earlier, the anodic fluorination of **1b** using Et_3_N·3HF provided α-fluorinated product **2b** selectively in good yield along with a small amount of fluorodesulfurization product **3b**. In contrast, the fluorodesulfurization reaction was significantly promoted with increasing HF content of the poly(HF) salts; especially the use of Et_3_N·5HF gave predominantly the fluorodesulfurization product **3b** in 85% yield (Figure 1a). Previously, we obtained **3b** in 75% yield by constant potential anodic oxidation of ethyl α-(phenylthio)acetate in a similar electrolytic solution [22]. A comparable dependency of product selectivity on supporting poly(HF) salts was also observed in a series of Et_4_NF·*n*HF (*n* = 3–5) although the product yields are moderate (Figure 1b).

According to these results, we carried out the anodic fluorination of other dithioacetals bearing different electron-withdrawing substituents such as acetyl, amide, and cyano groups under similar conditions. The results are summarized in Table 3. In the case of α,α-bis(phenylthio)acetone (**1d**) [32], the use of Et_3_N·3HF and Et_4_NF·3HF resulted in predominant α-fluorination to provide the corresponding monofluorinated product **2d** in good to moderate yields (Table 3, entries 1 and 3). On the contrary, when higher HF content salts such as Et_3_N·5HF and Et_4_NF·5HF were used, fluorodesulfurization product **3d** [22] was obtained almost exclusively in moderate or good yield (Table 3, entries 2 and 4). Regardless of poly(HF) salts, difluorinated product **4d** [33] was always formed due to the further oxidation of products **2d** and **3d**. In contrast, anodic fluorination of *N*,*N*-diethyl-α,α-bis(phenylthio)acetamide (**1f**) with Et_3_N·3HF required a large excess amount of electricity to consume the starting substrate **1f**, and fluorodesulfurization took place exclusively to provide the corresponding mono- and difluorinated products **3f** and **4f** [22,34] with the same ratio in rather low yields (Table 3, entry 5). The longer electrolysis caused the formation of complicated products. This result is quite different from the case of **1b** and **1d** (Table 2, entry 1 and Table 3, entry 1). Such different anodic behavior may be attributable to different p*K*_a_ values of the α-proton of the substrates. It is known that the acidity of the α-proton of *N*,*N*-diethylacetoamide is 4 to 5 times lower than that of acetone and ethyl acetate [35]. Therefore, the acidity of the α-proton of **1f** having an amide group would be much lower compared to that of **1b** and **1d** having an ester and acetyl group, respectively. Thus, it is reasonable that no deprotonation of the cationic intermediate of **1f** took place. Moreover, when a higher HF content poly(HF) salt like Et_3_N·5HF was used, fluorodesulfurization product **3f** was exclusively formed in good yield. This tendency is quite similar to the result of anodic fluorination of **1b** and **1d** (Figure 1a and Table 3, entries 2 and 4). Thus, it was found that due to the low acidity of the α-proton of **1f**, fluorodesulfurization took place prior to α-fluorination even in the presence of Et_3_N·3HF containing the free base Et_3_N. In sharp contrast to these cases, α,α-bis(phenylthio)acetonitrile (**1h**) [36] bearing a strongly electron-withdrawing cyano group underwent α-fluorination exclusively to produce α-fluorinated product **2h** in excellent yields regardless of the poly(HF) salts (Table 3, entries 7–12). In case of **1h**, no difluoro product was formed at all, which is probably due to a much higher oxidation potential of **2h** compared to that of the starting substrate **1h**. In support of this, we have already shown that introduction of one fluorine atom to the α-position of α-(phenylthio)acetonitrile increased the oxidation potential by 0.36 V [22].

**Table 3:** Anodic fluorination of dithioacetal derivative **1** in acetonitrile^a^.

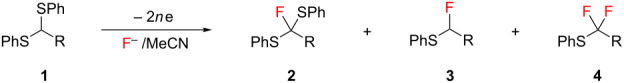						

Entry	R	Supporting electrolyte	Charge passed (F/mol)	Yield (%)^b,c^		

				**2**	**3**	**4**

1	COMe (**1d**)	Et_3_N·3HF	3.0	80 (66)	–	5
2	COMe (**1d**)	Et_3_N·5HF	2.5	–	63	3
3	COMe (**1d**)	Et_4_NF·3HF	2.7	60	–	10
4	COMe (**1d**)	Et_4_NF·5HF	2.5	6	78 (70)	1
5	CONEt_2_ (**1f**)	Et_3_N·3HF	5.0	0	18	17
6	CONEt_2_ (**1f**)	Et_3_N·5HF	3.0	0	72 (63)	1
7	CN (**1h**)	Et_3_N·3HF	3.0	98 (87)	0	0
8	CN (**1h**)	Et_3_N·4HF	2.8	98	0	0
9	CN (**1h**)	Et_3_N·5HF	2.5	90	0	0
10	CN (**1h**)	Et_4_NF·3HF	2.7	94	0	0
11	CN (**1h**)	Et_4_NF·4HF	2.5	95	0	0
12	CN (**1h**)	Et_4_NF·5HF	2.5	93	0	0

^a^Constant current (8 mA/cm^2^) electrolysis was carried out using 0.3 M supporting fluoride salt. ^b^Determined by ^19^F NMR. ^c^Isolated yields are given in parentheses.

**Scheme 3:**
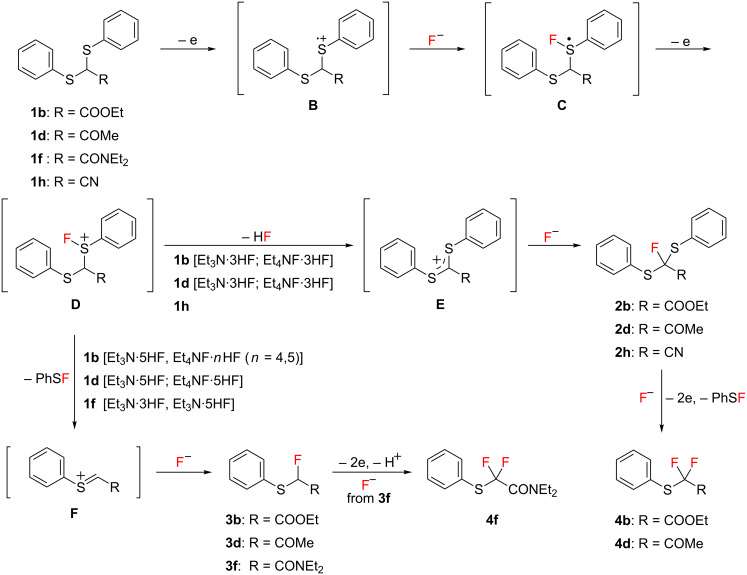
Plausible reaction mechanism for anodic fluorination of **1b**, **1d**, and **1f**.

A plausible mechanism for the anodic fluorination of dithioacetals **1b**, **1d**, and **1f** is shown in Scheme 3. The fluorination reaction is initiated by electron transfer from a sulfur atom of the substrate to generate the corresponding radical cation **B**, which traps a fluoride ion to afford radical **C**. This is followed by a further oxidation to give cationic intermediate **D**. In the cases of **1b** and **1d** having electron-withdrawing ester and acetyl groups, the α-protons are acidic enough and can be cleaved by either base, free Et_3_N (from Et_3_N·3HF) [37] or fluoride ions (from Et_4_NF·3HF). The resulting cation **E** reacts with a fluoride ion to form **2b** and **2d**. Further anodic fluorodesulfurization occurs to provide the corresponding difluorinated products **4b** and **4d**, respectively. On the other hand, the desulfurization of intermediate **D** followed by reaction with fluoride provides the corresponding fluorodesulfurization products **3b**, **3d**, and **3f**.

**Scheme 4:**
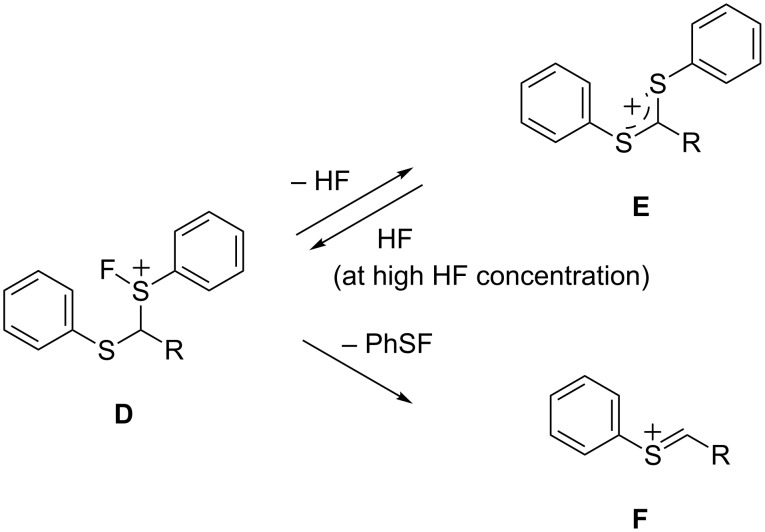
Mechanism for suppression of the elimination of HF (deprotonation) and preferable desulfurization of **D** at high concentrations of HF in an electrolytic solution.

When high HF content salts like Et_3_N·5HF and Et_4_NF·*n*HF (*n* = 4, 5) are used, the higher concentration of HF in the electrolytic solution would increase the amount of **D** rather than **E** in an equilibrium between them as shown in Scheme 4. Namely, the deprotonation of **D** would be retarded due to high concentration of protons in the solution, and consequently the C–S bond cleavage seems to take place more favorably than a deprotonation. A similar effect on the suppression of defluorination of CF_3_-enolate anion in the presence of a large amount of fluoride ions has been reported [38].

On the other hand, it is known that the acidity of α-protons of acetoamides is much lower compared to that of acetate and acetone as mentioned. Therefore, it is understandable that the anodic fluorination of **1f** having a weakly electron-withdrawing amide group resulted in fluorodesulfurization to provide **3f** even when Et_3_N·3HF containing free base, Et_3_N was used. As mentioned, the yield of monofluorodesulfurization product **3f** increased markedly by using high HF content salt, Et_3_N·5HF.

In sharp contrast, in the case of substrate **1h** having a cyano group, α-fluorination without desulfurization always took place even when Et_3_N·5HF and Et_4_NF·5HF were used. This can be explained in terms of fast deprotonation of cationic intermediate **D** promoted by a strongly electron-withdrawing cyano group as shown in Scheme 3.

## Conclusion

The regioselective anodic fluorination of ethyl α,α-bis(phenylthio)acetate and its acetone, acetoamide, and acetonitrile analogues was successfully carried out using various poly(HF) salts such as Et_3_N·*n*HF and Et_4_NF·*n*HF (*n* = 3–5) to provide α-fluoro and/or fluorodesulfurization products. The fluorinated product selectivity was found to depend on substituents and supporting poly(HF) salts. The unique product selectivity was tentatively explained in terms of electron-withdrawing ability (Taft σ*) of substituents and HF content of the used supporting poly(HF) salts.

## Supporting Information

File 1 General methods, synthetic procedures, characterzation data of all new compounds including copies of ^1^H NMR, ^13^C NMR and ^19^F NMR spectra.
