# Supplementary material for: Highly selective electrochemical fluorination of dithioacetal derivatives bearing electron-withdrawing substituents at the position α to the sulfur atom using poly(HF) salts
Source: Beilstein J Org Chem. 2015 Jan 19;11:85–91. doi: 10.3762/bjoc.11.12 (PMC4311748; doi:10.3762/bjoc.11.12)
Supplement: File 1 — General methods, synthetic procedures, characterzation data of all new compounds including copies of 1H NMR, 13C NMR and 19F NMR spectra. [file Beilstein_J_Org_Chem-11-85-s001.pdf]

Supporting Information

for

Highly selective electrochemical fluorination of dithioacetal derivatives bearing electron-withdrawing substituents at the position α to the sulfur atom using poly(HF) salts

Bin Yin, Shinsuke Inagi, Toshio Fuchigami*

Address: Department of Electronic Chemistry, Tokyo Institute of Technology, Nagatsuta, Midori-ku, Yokohama 226-8502, Japan

Email: Toshio Fuchigami - fuchi@echem.titech.ac.jp

*Corresponding author

General methods, synthetic procedures, characterzation data of all new compounds including copies of ^{1}H NMR, ^{13}C NMR and ^{19}F NMR spectra

Table of Contents

General Information S2

General Procedure for the preparation of α,α-bis(phenylthio) derivatives **1b**, **1d**, **1f**, and **1h** S2

Typical anodic fluorination of α,α-bis(phenylthio) derivatives S4

^{1}H, ^{13}C, and ^{19}F NMR spectra for the new compounds S6

General Information

^{1}H NMR, ^{19}F NMR, and ^{13}C NMR spectra were recorded at 270, 254, and 68 MHz, respectively using a JEOL JNM EX-270 spectrometer (270.05 MHz) in a deuteriochloroform ($CDCl_3$) solution containing tetramethylsilane (TMS, 0.00 ppm), monofluorobenzene (C_6H_5F, –36.5 ppm) and $CDCl_3$ (77.0 ppm) as internal standards. Purification of the fluorinated products was achieved by flash chromatography on Nacalai Tesque Silica Gel 60 (spherical, neutrality) or by high performance liquid chromatography (HPLC) performed on a Shiseido Superiorex ODS column (20 mm id x 250 mm, MeCN). Mass spectra were obtained by EI method with a Shimadzu GCMS-QP5050A. High resolution mass spectra (HRMS) were taken on a JEOL JMS-700 or Bruker Daltonics microTOF II mass spectrometer. Elemental analysis was performed on LECO CHNS-932 VTF-90 (C, H) and J-Science HSU-20 (F). Cyclic voltammetry was performed with an ALS CH instrument electrochemical analyzer model 600C. Electrolysis experiments were carried out using a Metronix Corp. constant current power supply model 5944 and were monitored with a Hokuto Denko Coulomb/Amperehour Meter HF-201.

General Procedure for preparation of α,α-bis(phenylthio) derivatives 1b, 1d, 1f, and 1h

To a stirred solution of ethyl α-(phenylthio)acetate (**1a**, 30 mmol) in CCl_4 (50 ml) containing benzoyl peroxide (1.5 mmol, 5 mol %), was added *N*-bromosuccinimide (NBS, 30 mmol, 1.0 equiv). The reaction mixture was refluxed until the starting material was consumed (monitored by TLC) and then left to cool to room temperature. The crude product was dissolved in anhydrous diethyl ether (100 ml). To this solution was added sodium hydride (30 mmol, 1.0 equiv), then left it stirring at 0 ^{o}C. After 10 min, thiophenol (30 mmol, 1.0 equiv) was added dropwise into the reaction mixture and then slowly allowed to warm to room temperature. After stirring overnight, the reaction mixture was filtrated to remove insoluble sodium bromide and the filtrate was evaporated under vacuum. The oily crude product was purified by column chromatography on silica gel using ethyl acetate/hexane as an eluent to give pure ethyl α,α-bis(phenylthio)acetate (**1b**) (63%). α,α-Bis(phenylthio)acetone (**1d**)

(66%), α,α-bis(phenylthio)acetonitrile (**1h**) (29%) were prepared similarly from phenylthioacetone (**1c**) and phenylthioacetonitrile (**1g**), respectively. Compounds **1b, 1d**, **1h** were identified by comparing the spectra with those of the authentic samples [1-4].

N,*N*-Diethyl-α,α-bis(phenylthio)acetamide (**1f**) was prepared from **1b** as follows. To a solution of dithioacetal ester **1b** (10 mmol) in THF (50 ml) was added 10% NaOH solution. After stirring for 2 h, THF was removed under reduced pressure. The residue was poured into water (20 ml) and extracted with Et_2O. The aqueous layer was combined and acidified (to pH 1, monitored by pH test paper) with 1 N HCl. Then, the acidic product was extracted with AcOEt (x 3). The combined extracts were dried over sodium sulfate followed by filtration. After evaporation of the solvent, the corresponding acid was obtained in 86% yield. The acid was converted to its acid chloride by treatment with thionyl chloride (25 mmol, 2.5 equiv) in $CHCl_3$ (10 ml). Excess thionyl chloride was removed under vacuum and the residue was dissolved in 30 ml of $CHCl_3$. Diethylamine (20 mmol, 2.0 equiv) was added into the solution. After stirring for 1 h at room temperature, $CHCl_3$ was removed under reduced pressure. The crude product was purified through column chromatography on silica gel using ethyl acetate/hexane as eluent to give pure amide **1f** in 64% yield**.**

***N*,*N*-Diethyl-α,α-bis(phenylthio)acetamide (1f)**

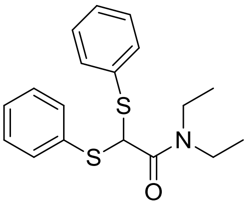

1f

Colorless crystals: mp 37-38 °C; ^{1}H NMR (270 MHz, $CDCl_3$) δ ppm 1.05 (t, 3H, *J* = 8.1 Hz), 1.10 (t, 3H, *J* = 8.1 Hz), 3.23 (q, 2H, *J* = 7.1 Hz), 3.29 (q, 1H, *J* = 7.1 Hz), 5.07 (s, 1H), 7.32 (dd, 4H, *J* = 6.8, 3.8 Hz), 7.31 (dd, 1H, *J* = 3.8, 3.0 Hz), 7.29-7.35 (m, 1H), 7.51 (t, 1H, *J* = 1.1 Hz), 7.53 (t, 1H, *J* = 1.4 Hz), 7.54 (dd, 1H, *J* = 2.7, 1.6 Hz), 7.49-7.57 (m, 1H); ^{13}C NMR (68 MHz, $CDCl_3$) δ ppm 12.69, 14.40, 40.83, 42.33, 58.76, 128.54, 128.91, 132.94, 133.77, 166.67; HRMS (ESI): *m/z* $[M+Na]^+$ Calcd for $C_{18}H_{21}NOS_2Na$: 354.0962; Found: 354.0957. HRMS (APCI): *m/z* $[M+H]^+$ Calcd for $C_{18}H_{22}NOS_2$: 332.1143; Found: 332.1137.

Typical anodic fluorination of α,α-bis(phenylthio) derivatives 1

In a similar manner to a procedure that has been reported previously [5], the anodic fluorination of dithioacetal derivatives was carried out as follows: Anodic fluorination of **1** (0.1 mmol) was carried out with platinum plate electrodes (1 x 1 cm^2) in 0.3 M Et_3N-3HF/MeCN (2 ml) in an undivided cell under nitrogen atmosphere at room temperature. A constant current (8 mA/cm^2) was passed until the starting material was mostly consumed (monitored by TLC and GC–MS). After the electrolysis, the electrolytic solution was passed through a short column of silica gel using ethyl acetate to remove excess fluoride salts. The eluent was evaporated under reduced pressure, and the residue was further purified by column chromatography on silica gel using ethyl acetate/hexane (15/1, v/v) as an eluent. Fluorinated products **3b**, **3d**, **3f**, **4b**, **4d**, **4f** were identified by ^{19}F NMR and GC–MS spectra [6-9].

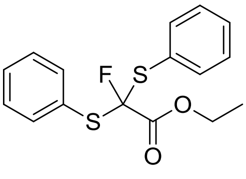

2b

Ethyl α-fluoro-α,α-bis(phenylthio)acetate (2b)

Colorless oil; ^{1}H NMR (270 MHz, $CDCl_3$) δ ppm 1.03 (t, 3H, *J* = 7.0 Hz), 3.97 (q, 2H, *J* = 7.1 Hz), 7.33 (dd, 1H, *J* = 3.8, 2.2 Hz), 7.36 (d, 1H, *J* = 7.0 Hz), 7.36 (dd, 1H, *J* = 8.1, 3.8 Hz), 7.38 (dd, 1H, *J* = 10.5, 5.9 Hz), 7.41 (dd, 1H, *J* = 6.8, 4.3 Hz), 7.44 (t, 1H, *J* = 1.4 Hz), 7.59 (dd, 1H, *J* = 8.1, 0.5 Hz), 7.59 (dd, 1H, *J* = 7.8, 0.5 Hz), 7.59-7.62 (m, 1H); ^{19}F NMR (254 MHz, $CDCl_3$) δ ppm -29.30 (s. 1F); ^{13}C NMR (68 MHz, $CDCl_3$) δ ppm 13.79, 62.93, 107.09 (d, C-F, *J* = 279.3 Hz), 129.01, 130.13, 136.02 (d, *J* = 1.4 Hz), 128.39 (d, *J* = 1.4 Hz), 133.36, 164.83 (d, *J* = 33.2 Hz); GCMS: *m/z* = 322 $[M]^+$, 231, 195, 149, 121, 109, 77; HRMS (ESI): *m/z* $[M+Na]^+$ Calcd for $C_{16}H_{15}FO_2S_2Na$: 345.0395; Found: 345.0390; HRMS (APCI): *m/z* $[M-F]^+$ Calcd for $C_{16}H_{15}O_2S_2$: 303.0513; Found: 303.0508.

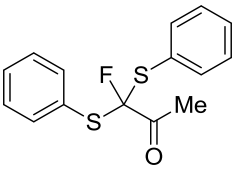

2d

α-Fluoro-α,α-bis(phenylthio)acetone (2d)

Colorless oil; ^{1}H NMR (270 MHz, $CDCl_3$) δ ppm 2.43 (d, 3H, *J* = 11.9 Hz), 7.29-7.37 (m, 4H) 7.43-7.47 (m, 2H), 7.50-7.54 (m, 3H); ^{19}F NMR (254 MHz, $CDCl_3$) δ ppm -32.95 (s, 1F); ^{13}C NMR (68 MHz, $CDCl_3$) δ ppm 26.09, 111.06 (d, C-F, *J* = 283.3 Hz), 128.03 (d, C-S, *J* = 1.1 Hz), 129.24, 130.14, 135.74, 197.32 (d, *J* = 30.1 Hz); GCMS: *m/z* = 273 $[M-F]^+$, 230, 164, 109, 77; HRMS (FAB): *m/z* $[M+Na]^+$ Calcd for $C_{15}H_{13}FOS_2Na$:

315.0290; Found: 315.0294.

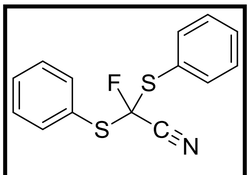
2h

α-Fluoro-α,α-bis(phenylthio)acetonitrile (2h)

Colorless crystal: mp 51-52 °C; ^{1}H NMR (270 MHz, $CDCl_3$) δ ppm 7.41-7.55 (m, 6H), 7.67-7.70 (m, 4H); ^{19}F NMR (254 MHz, $CDCl_3$) δ ppm -18.99 (s, 1F); ^{13}C NMR (68 MHz, $CDCl_3$) δ ppm 99.01 (d, C-F, *J* = 267.7 Hz), 112.64 (d, CN, *J* = 49.6 Hz), 127.16, 129.58, 131.29, 136.62; GCMS: *m/z* = 275 $[M]^+$, 166, 109, 77; HRMS (EI): *m/z* $[M]^+$ Calcd for $C_{14}H_{10}FNS_2$: 275.0239; Found: 275.0234.

References

1. Tabti, B.; Gourmala, C.; Bounouara Bouderba, H.; Mulengi, J. K. *J. Soc. Alger. Chim.* **1996**, *6*, 199–206.

2. Ritter, R. H.; Cohen, T. *J. Am. Chem. Soc.* **1986**, *108*, 3718–3725. doi: 10.1021/ja00273a028

3. Keiko, N. A.; Funtikova, E. A.; Stepanova, L. G.; Chuvashev, Y. A.; Larina, L. I. *Russ. J. Org. Chem.* **2002**, *38*, 970–976. doi: 10.1023/A:1020893310626

4. Ishibashi, H.; Okada. M.; Sato, K.; Ikeda, M.; Ishiyama, K.; Tamura, Y. *Chem. Pharm. Bull.* **1985**, *33*, 90–95. doi: 10.1248/cpb.33.9

5. Shaaban, M. R.; Ishii, H.; Fuchigami, T. *J. Org. Chem.* **2000**, *65*, 8685–8689. doi: 10.1021/jo001129u

6. Fuchigami, T.; Shimojo, M.; Konno, A. *J. Org. Chem.* **1995**, *60*, 3459–3464. doi: 10.1021/jo00116a037

7. Yagupolski, L.; Korinko, V. A. *Zh. Obshch. Khim.* **1969**, *39*, 1747.

8. Gouault, S.; Guérin, C.; Lemoucheux, L.; Lequeux, T.; Pommelet, J. C. *Tetrahedron Lett.* **2003**, *44*, 5061–5064. doi: 10.1016/S0040-4039(03)01134-1

9. Konno, A.; Fuchigami, T. *J. Org. Chem.* **1997**, *62*, 8579-8581. doi: 10.1021/jo971248i

^{1}H, ^{13}C , and ^{19}F NMR spectra for the new compounds

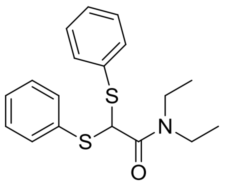

1f

N,N-Diethyl-α,α-bis(phenylthio)acetamide

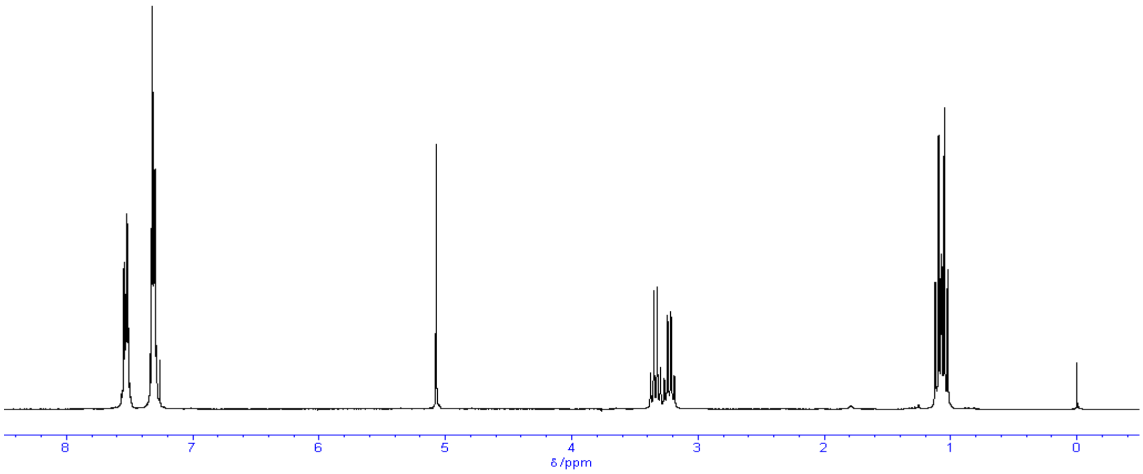

Figure S1. ^{1}H NMR Spectrum of **1f**.

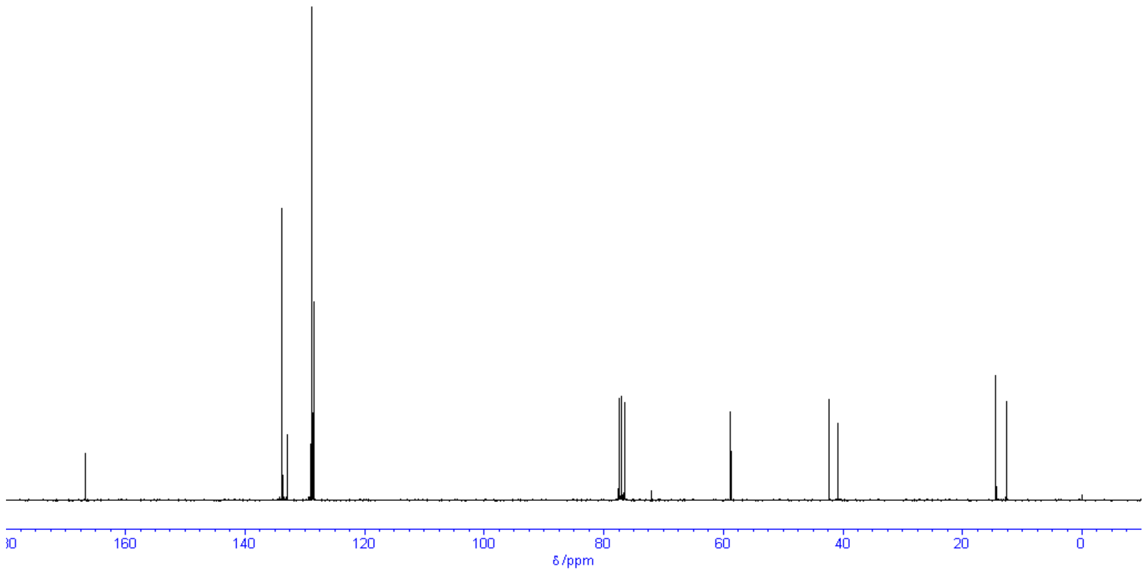

Figure S2. ^{13}C NMR Spectrum of **1f**.

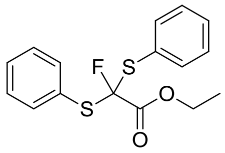

2b

Ethyl α-fluoro-α,α-bis(phenylthio)acetate

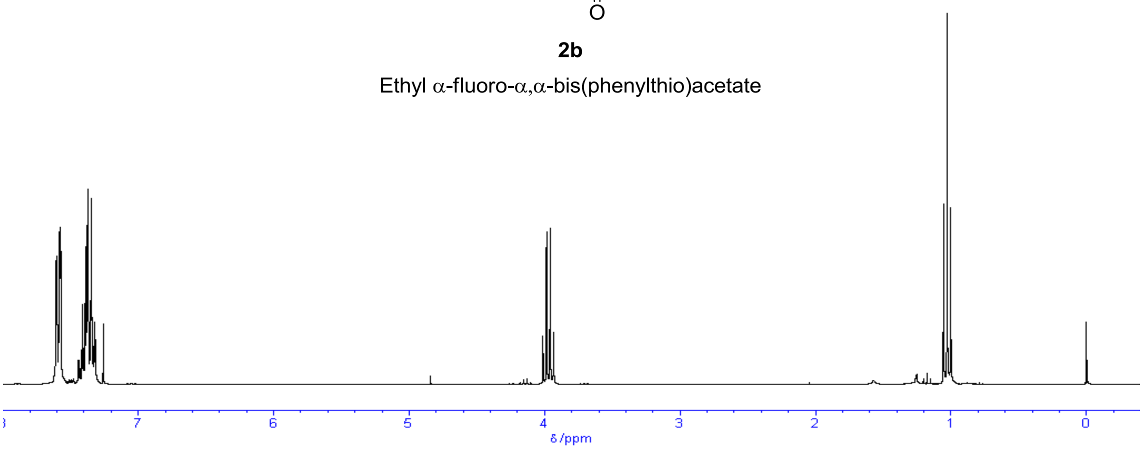

Figure S3. ^{1}H NMR Spectrum of **2b**.

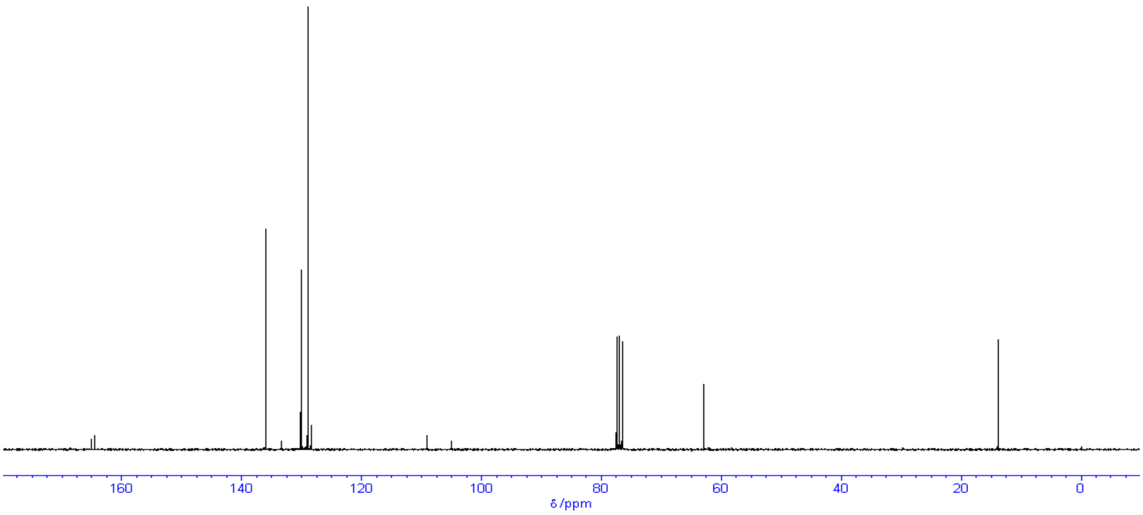

Figure S4. ^{13}C NMR Spectrum of **2b**.

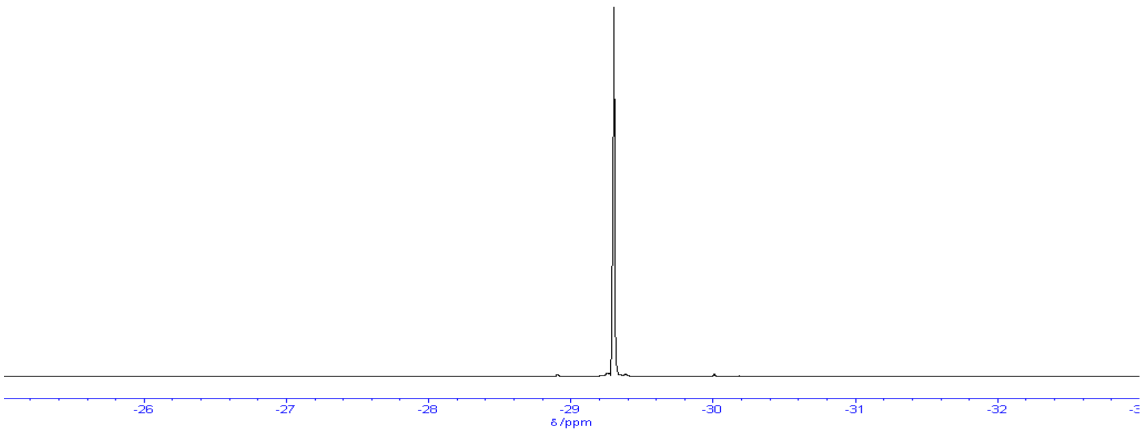

Figure S5. ^{19}F NMR Spectrum of **2b**.

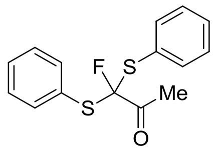

2d

α-Fluoro-α,α-bis(phenylthio)acetone

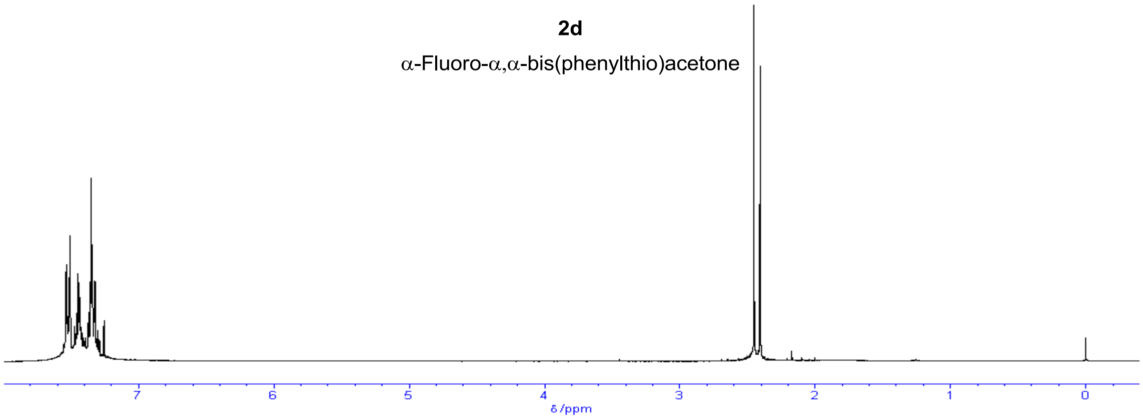

Figure S6. ^{1}H NMR Spectrum of **2d**.

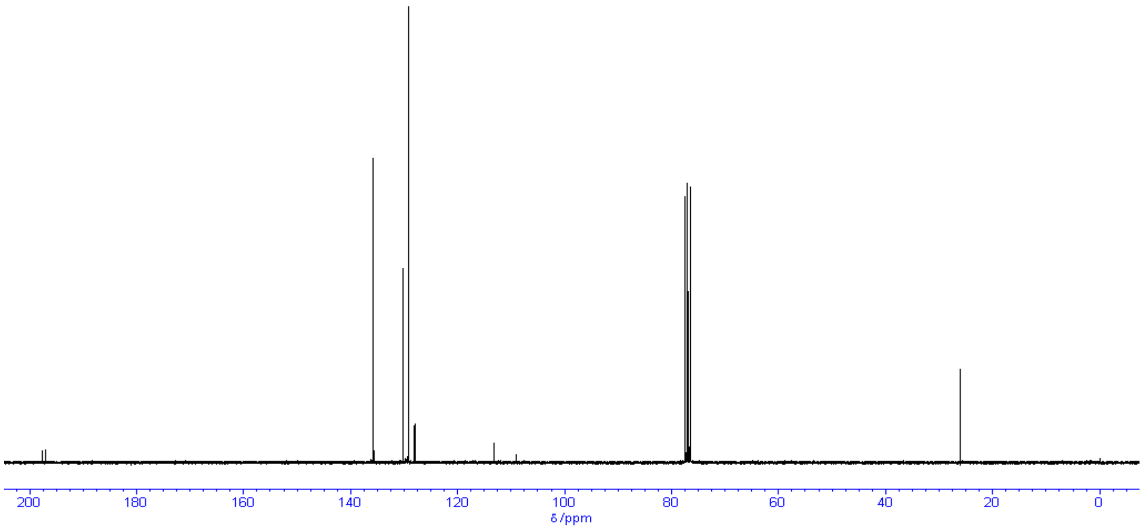

Figure S7. ^{13}C NMR Spectrum of **2d**.

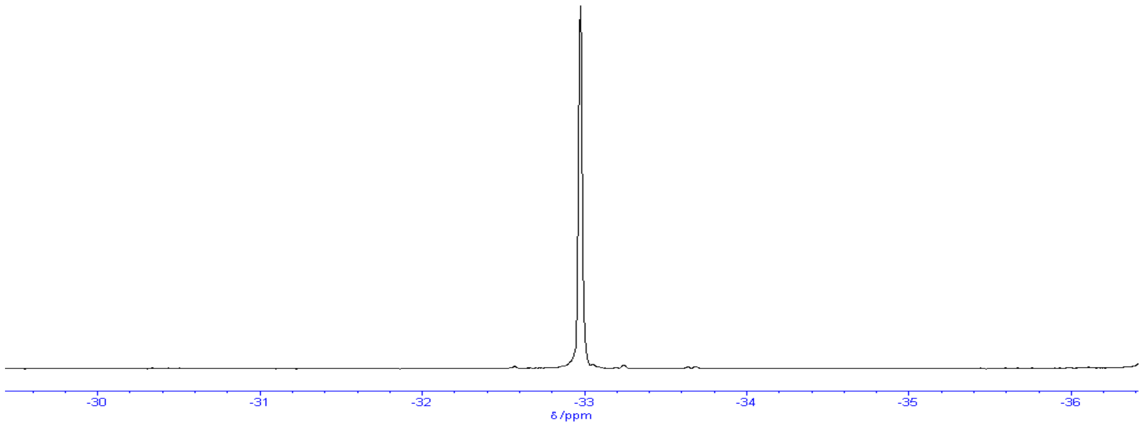

Figure S8. ^{19}F NMR Spectrum of **2d**.

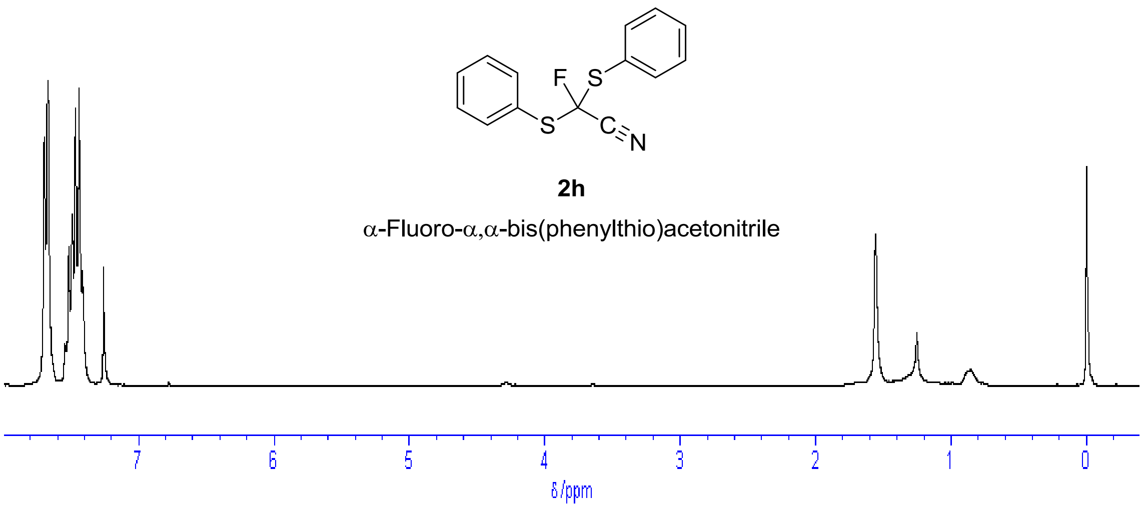

Figure S9. ^{1}H NMR Spectrum of **2h**

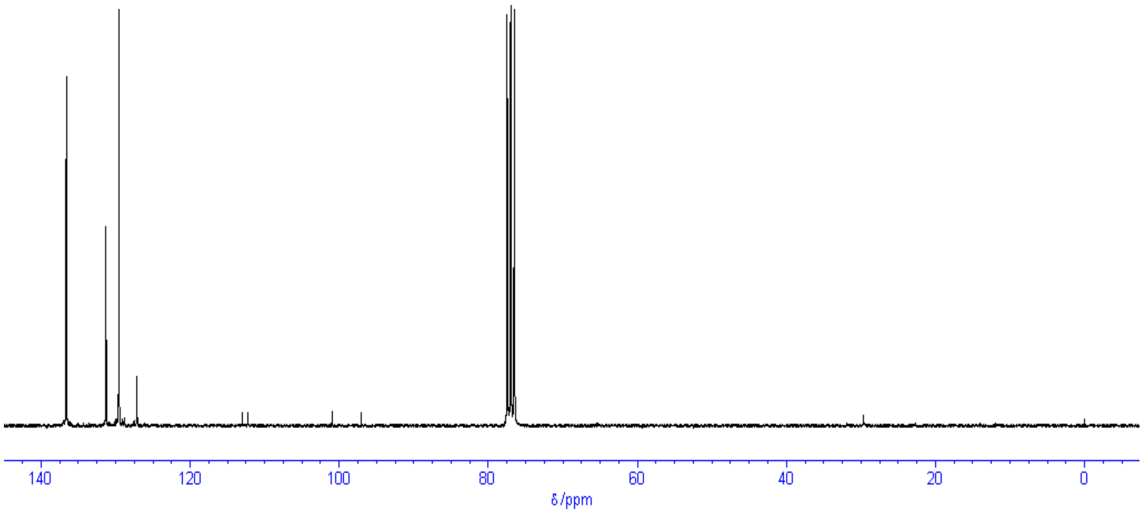

Figure S10. ^{13}C NMR Spectrum of **2h**.

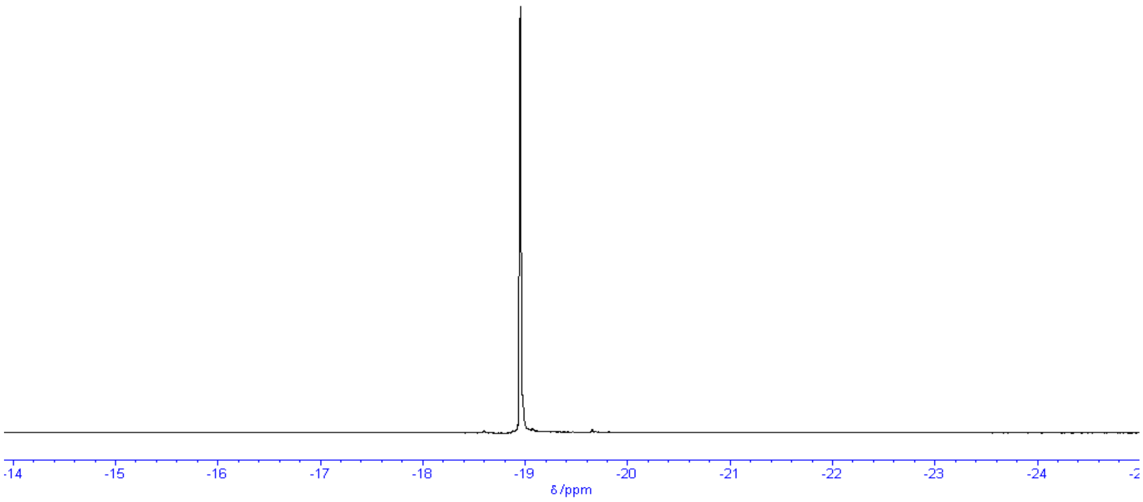

Figure S11. ^{19}F NMR Spectrum of **2h**.
